# Different *Cutibacterium acnes* Phylotypes Release Distinct Extracellular Vesicles

**DOI:** 10.3390/ijms23105797

**Published:** 2022-05-21

**Authors:** Anna Chudzik, Paweł Migdał, Mariola Paściak

**Affiliations:** Hirszfeld Institute of Immunology and Experimental Therapy, Polish Academy of Sciences, 53-114 Wroclaw, Poland; anna.chudzik@hirszfeld.pl (A.C.); pawel.migdal@hirszfeld.pl (P.M.)

**Keywords:** extracellular vesicles, microvesicles, *Cutibacterium acnes*, lipid analysis, protein analysis, SDS-PAGE, TLC, MALDI-TOF MS, TEM

## Abstract

Bacterial extracellular vesicles (EVs) perform various biological functions, including those that are critical to microbes. Determination of EVs composition allows for a deep understanding of their role in the bacterial community and communication among them. *Cutibacterium acnes*, formerly *Propionibacterium* *acnes*, are commensal bacteria responsible for various infections, e.g., prosthesis, sarcoidosis, soft-tissue infections, and the most known but still controversial—acnes lesion. In *C. acnes*, three major phylotypes represented variable disease associations. Herein, for the first time, we present a comparative analysis of EVs obtained from three *C. acnes* phylotypes (IA1, IB, and II) to demonstrate the existence of differences in their protein and lipid composition. In the following work, the morphological analysis of EVs was performed, and the SDS-PAGE protein profile and the lipid profile were presented using the TLC and MALDI-TOF MS methods. This study allowed us to show major differences between the protein and lipid composition of *C. acnes* EVs. This is a clear indication that EVs released by different phylotypes of the one species are not identical to each other in terms of composition and should be separately analyzed each time to obtain reliable results.

## 1. Introduction

Bacterial extracellular vesicles (EVs) have recently gained a special interest in the scientific community, both due to their therapeutic potential and participation in intercellular communication [1,2]. Secreted from the bacterial cells extracellular vesicles are spherical nanostructures with dimensions ranging from 20 to 300 nm. Formerly, they are disregarded as artifacts of bacterial growth, bubbles, or rubbish. It is now understood that EVs are purposely secreted by bacteria to aid in communication and contribute to numerous bacterial functions. Although the mechanisms controlling the release and selection of contents remain unexplained, reports indicate their important role in the bacterial community, including protective functions, support in obtaining nutrients, and horizontal gene transfer. EVs, apart from intracellular communication, take part in the pathogenesis and regulation of host immunity [3]. Due to their structure consisting of a lipid bilayer, they may contain various lipids but also numerous proteins, cellular metabolites, and nucleic acids [4]. It is the proteins and lipids that can be important elements of cell-to-cell communication via EVs. Composition analysis would improve the understanding of their function in the bacterial environment and the interaction between EVs and eukaryotic cells [5,6,7]. Careful elaboration of the composition will not only clarify their biological significance but also make it possible to determine their usefulness in medical applications. Outer membrane vesicles (OMVs) have been used successfully in the design of vaccines, and liposome constructs have found use as carriers for therapeutic substances [8,9]. Hence, further research into the composition, functions, and interactions in which bacterial EVs participate is necessary.

*Cutibacterium acnes* (formerly *Propionibacterium acnes*) is a Gram-positive aero-tolerant anaerobic bacterium, which is a part of the human microbiota and is a major cultivable organism found on the human skin. A study of the human microbiome using DNA sequencing confirms that *Cutibacterium* belongs to abundant resident bacterial genera on the surface layers of the human skin; in human follicles, *C. acnes* is often the sole bacterial inhabitant of such areas [10]. These bacteria are successful colonizers of different human tissue sites, and such ubiquitous prevalence might indicate that the human host benefits from their presence.

*Cutibacterium acnes* are associated with a common skin disorder—acne vulgaris [11], but its role is controversial and has not been confirmed [12]. *C. acnes* belongs to opportunistic bacteria responsible for post-operative infections and biofilm-associated infections related to indwelling medical devices. They were isolated from prosthetic joint infections, osteomyelitis, endocarditis, endophthalmitis, and SAPHO syndrome characterized by inflammatory bone disorders [13].

Even though *C. acnes* is numerous both on the skin surface of healthy people and of those with acne vulgaris, studies have shown they differed with regard to the presence of various phylotypes [14]. There are three types within the species *C. acnes*, recently assigned to novel subspecies: I—*C. acnes* subsp. *acnes*, II—*C. acnes* subsp. *defendens* [15], III—*C. acnes* subsp. *elongatum* [16,17]. The above taxonomy is based primarily on genomic analysis and lipase activity in the individual types. Based on multilocus sequence typing, *C. acnes* strains are classified further into six main phylotypes, designated IA1, IA2, IB, IC, II, and III [17,18].

Literature data indicate that *C. acnes* phylogroups are associated with different clinical conditions [19], e.g., the type IA1 group dominated in isolates cultured from acne-affected regions, and type IB was found in isolates from soft tissue infections [19]. *C. acnes* isolated from prostatic tissues obtained after prostatectomy and positively associated with prostatic inflammation represent *C. acnes* phylotype II [20]. Comparative studies analyzing the properties of individual phylotypes within the *C. acnes* species have already been carried out [21]; however, a similar comparison regarding the EVs released by different *C. acnes* phylotypes, according to our knowledge, have not been performed yet.

EVs secreted by *C. acnes* type I were first reported in 2017 [11]. Next, Choi and collaborators found that *C. acnes* derived EVs can induce an acne-like phenotype in keratinocytes and reconstituted human skin models [22]. However, it is not known how *C. acnes* communicate with host cells after infection and neither the role of lipids present in EV in these interactions is recognized. Moreover, information concerning EVs of other representatives of *Cutibacterium* phylotypes is missing.

In this work, we intend to perform a comparative analysis of EVs obtained from three different *C. acnes* phylotypes to characterize EVs protein and lipid content and compare their morphology.

## 2. Results

### 2.1. Morphological Characteristics of Cutibacterial EVs

EVs derived from *Cutibacterium acnes* were purified from culture supernatant according to the previously established protocol for Gram-positive bacterial EVs [23] with modifications. For bacteria cultivation, we used thioglycollate-soy broth medium (TS) and anaerobic conditions.

The described method of culture allowed us to obtain satisfactory amounts of bacterial extracellular vesicles for further analysis. From 2 L of bacterial culture, 2.092 mg/mL EVs of *C. acnes* DSM 16379, 1.355 mg/mL EVs of *C. acnes* DSM 1897, and 2.994 mg/mL EVs of *C. acnes* PCM 2334 were obtained, respectively, in terms of proteins.

**Figure 1:**
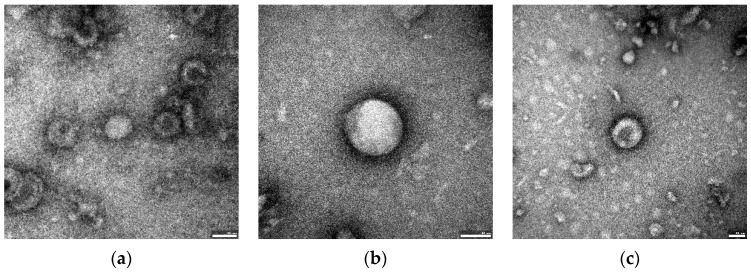
Transmission electron microscopy of *Cutibacterium acnes* EVs. (**a**) EVs isolated from *C. acnes* DSM 1897, type IA1 (**b**) EVs isolated from *C*. *acnes* DSM 16379, type IB (**c**) EVs isolated from *C. acnes* PCM 2334, type II. Bar 50 nm.

The homogeneity and purity of the EVs fractions were verified using transmission electron microscopy (TEM) and Dynamic Light Scattering (DLS). TEM images of the individual *Cutibacterium acnes* EVs obtained were presented in Figure 1a–c.

**Figure 2:**
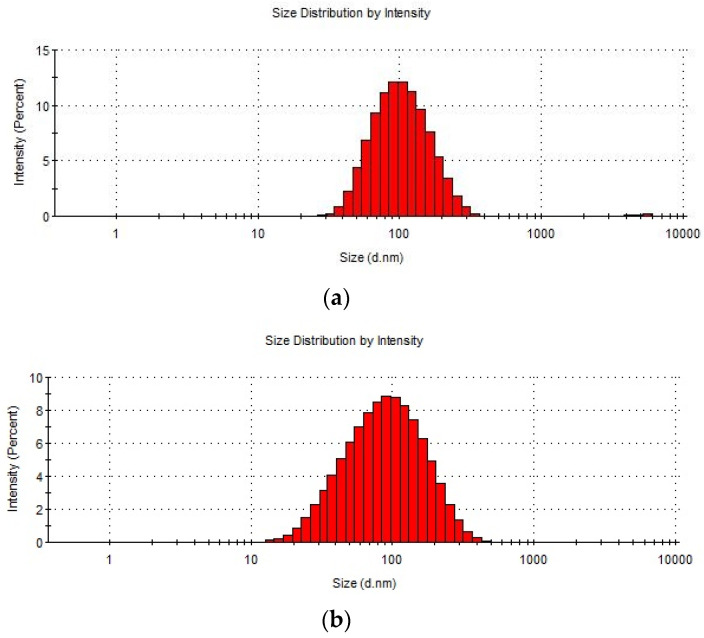

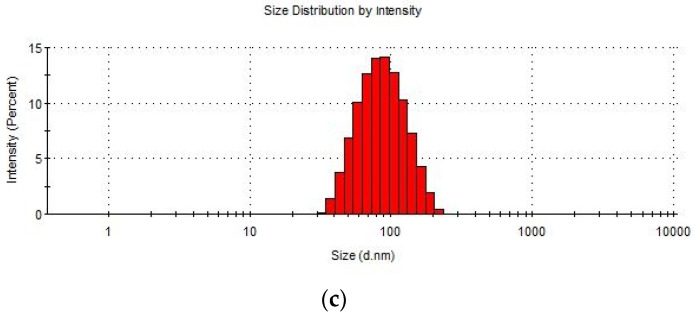
Size distribution of *Cutibacterium acnes* EVs. (**a**) *C. acnes* EVs isolated from DSM 1897 type IA1, (**b**) EVs isolated from *C. acnes* DSM 16379 type IB, (**c**) EVs isolated from *C. acnes* PCM 2334 type II.

The mean size of the diameter of the acquired EVs for individual *C. acnes* strains determined by DLS were 93.84 nm (±52.29) for DSM 1897 (Figure 2a), 83.20 nm (±52.01) for DSM 16379 (Figure 2b), and 80.15 nm (±35.55) for PCM 2334 (Figure 2c).

### 2.2. Protein Profile of C. acnes Cell Fractions and Isolated EVs

**Figure 3:**
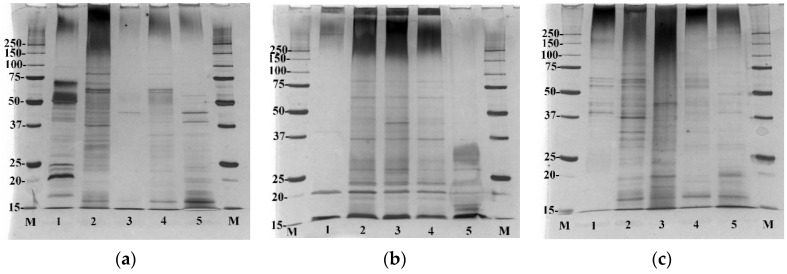
SDS-PAGE of EVs (1), whole-cell lysate (2), surface proteins (3), cytosolic proteins (4), cell wall proteins (5). (**a**) *C. acnes* DSM 1897 type IA1, (**b**) *C. acnes* DSM 16379 type IB, (**c**) *C. acnes* PCM 2334 type II. Silver staining.

Sodium dodecyl sulphate polyacrylamide gel electrophoresis (SDS-PAGE) was performed for the *C. acnes* cellular protein fractions and EVs fractions released by each *C. acnes* strain (Figure 3). No contaminants from the culture medium were present in the SDS-PAGE protein fractions (Figure S1). This allowed for the comparison of proteins present in EVs against cell fractions and against each phylotype.

This analysis revealed clear differences between the proteins found in EVs obtained from each *C. acnes* strain. In the case of EVs isolated from *C. acnes* type IB, only a few proteins are observed, and they can be found in each cellular fraction of *C. acnes* DSM 16379 (Figure 3b). EVs from type IA1 (DSM 1897) showed the presence of numerous vesicle proteins, and some of them were also found in the fractions of whole cell lysate, cytosolic proteins, and cell wall proteins of *C. acnes* DSM 1897 (Figure 3a). Released *C. acnes* type II EVs proteins were also found in protein fractions isolated from this strain, especially the cytosolic proteins (Figure 3c). The comparison of individual vesicle fractions allows for the demonstration of significant differences, especially in the size and abundance of the proteins. EVs released by the *C. acnes* DSM 16379 strain were characterized by the poorest protein profile—the presence of proteins in the range of about 20 kDa was found. The most abundant proteins were EVs released by the DSM 1897 strain (type IA1), with numerous proteins in the range of 15–75 kDa. Despite the fact that all phylotypes belong to the same species and were processed in identical culture and isolation conditions, the protein profiles may represent differences in the adaptation of these strains to environmental conditions.

### 2.3. Lipid Profiles of EVs and Whole-Cell Extracts

**Figure 4:**
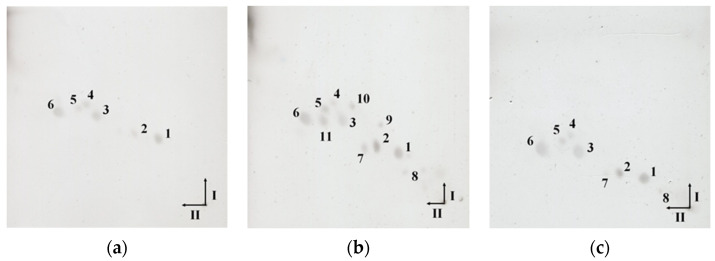
2D-TLC lipid profiles of EVs: (**a**) *C. acnes* DSM 1897 type IA1, (**b**) *C. acnes* DSM 16379 type IB, and (**c**) *C. acnes* PCM 2334 type II. The black arrows (I and II) indicate the directions in which the chromatograms were developed. The lipids visible on the plates are numbered in the range 1–11. The TLC was developed in (I) chloroform-methanol-water (65:25:4, *v*/*v*), II chloroform-methanol-acetic acid-water (80:15:12:4, *v*/*v*), and for the detection of lipids, copper sulfate reagent and heating at 130 °C were applied.

To confirm the lipid presence in isolated EVs, lipid extracts obtained by the Bligh-Dyer method were analyzed by two-dimensional thin-layer chromatography (2D-TLC). The spots visible on the TLC plates correspond to the individual lipids present in the obtained lipid extracts (Figure 4). The differences between the lipid composition of EVs from individual strains are clearly visible. Lipid extract of EVs obtained from *C. acnes* DSM 1897 contains only six lipids (Figure 4a), while *C. acnes* PCM 2334 (Figure 4c) and DSM 16379 (Figure 4b) contain 8 and 11 lipids, respectively. The use of copper sulfate acid reagent allows for the visualization of polar lipids—both phospholipids and glycolipids. Lipids numbered 1–6 were present in extracts obtained from each phylotype. Worth mentioning are the attempts to use different reagents to visualize phospho- and glycolipids, i.e., Dittmer & Lester reagent and orcinol, respectively, were unsuccessful. In the culture medium after lipid extraction, no lipid spots were visible in 2D-TLC (Figure S2).

**Figure 5:**
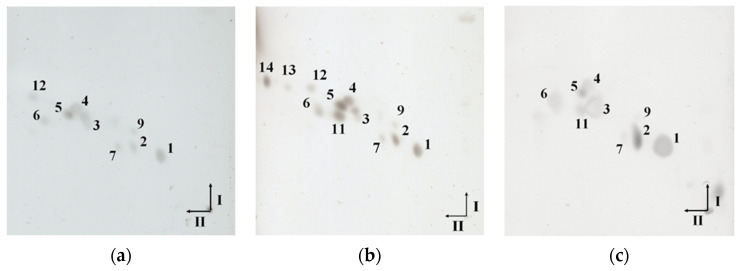
2D-TLC lipid profiles of the whole-cell extracts obtained from (**a**) *C. acnes* DSM 1897 type IA1, (**b**) *C. acnes* DSM 16379 type IB, and (**c**) *C. acnes* PCM 2334 type II. The black arrows (I and II) indicate the directions in which the chromatograms were developed. The lipids visible on the plates are numbered in the range 1–14. The TLC was developed in the solvent system (I) chloroform-methanol-water (65:25:4, *v*/*v*), then in (II) chloroform-methanol-acetic acid-water (80:15:12:4, *v*/*v*) and for the detection of lipids copper sulfate reagent and heating at 130 °C were applied.

Similar to the case of EVs lipid extracts, cellular lipid extracts differ from one another depending on the strain (Figure 5). In lipid extracts obtained from *C. acnes* DSM 1897 (Figure 5a) and PCM 2334 (Figure 5c), nine lipid compounds were present, while from *C. acnes* DSM 16379 (Figure 5b)—twelve (the most abundant lipid profile). Compared to vesicular lipid extracts, whole-cell extracts were more abundant in lipids, confirming that the selection of EVs composition is regulated by bacterial cells. These extracts differ not only in the number of lipids—the cellular lipids extract did not contain lipids number 8 and 10 in its profile but had additionally three lipids compounds (Nos. 12, 13, and 14) (Figure 5b). TLC analysis of cellular lipids of *C. acnes* strain 1897 showed the presence of nine lipids in the whole-cell extract (Figure 5a), while the vesicular extract contained six lipids (Figure 4a). These observations indicate the selectivity of the bacterial strain in terms of the selection of lipids secreted in the form of EVs.

### 2.4. MALDI-TOF MS Lipid Profiling

**Figure 6:**
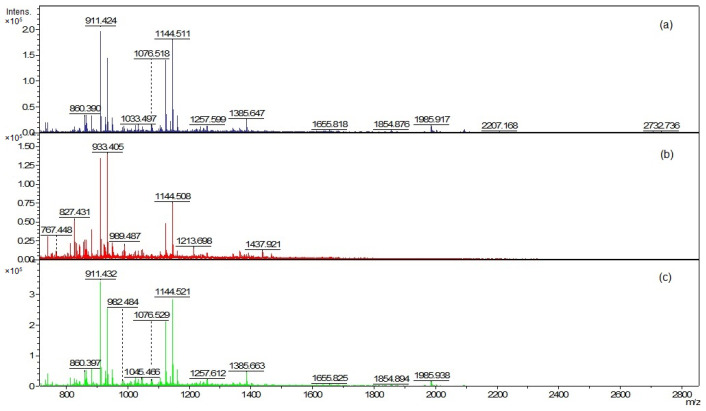
Positive ion MALDI-TOF MS spectra of EVs lipids (**a**) *C. acnes* DSM 1897 type IA1, (**b**) *C. acnes* DSM 16379 type IB, and (**c**) *C. acnes* PCM 2334 type II.

The EVs and cellular lipid extracts were also analyzed using matrix-assisted laser desorption ionization–time of flight mass spectrometry (MALDI-TOF MS) (Figure 6 and Figure 8). The mass spectra were acquired in the positive ion mode due to the much higher quality of the spectra than in the negative ion mode.

**Figure 7:**
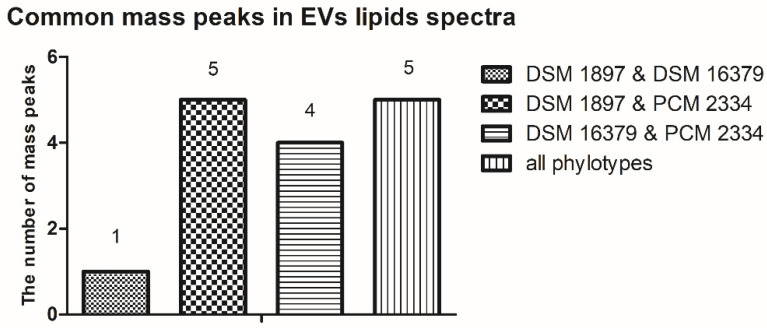
Comparison of the presence of common mass peaks of the vesicular lipids for the different *C. acnes* strains in MALDI-TOF MS analysis.

The mass peaks list generated using the FlexAnalysis software allows for a more accurate comparison of vesicle lipid profiles of different *C. acnes* phylotypes (Tables S1–S3). In all analyzed EV lipid extracts, there are five common mass peaks (739.4 *m*/*z*, 755.4 *m*/*z*, 827.4 *m*/*z*, 1341.8 *m*/*z*, 1363.8 *m*/*z*) (Figure 6 and Figure 7, Tables S1–S3). One common mass peak (767.4 *m*/*z*) was found for the EVs from *C. acnes* DSM 16379 and 1897 strains (Figure 7). For EVs from *C. acnes* DSM 1897 and PCM 2334 strains, five common mass peaks were found (998.4 *m*/*z*, 1010.5 *m/z*, 1655.8 *m*/*z*, 1854.8 *m*/*z*, 2093.1 *m*/*z*), and a comparison of the EVs from *C. acnes* DSM 16379 and PCM 2334 strains led to the finding of four common mass peaks (812.3 *m*/*z*, 834.3 *m*/*z*, 1023.4 *m*/*z*, 1045.4 *m*/*z*) (Figure 7). The comparison of the presence of mass peaks in the extracts was made after removing the peaks present in the matrix or the culture medium (Tables S7 and S8, Figure S3).

**Figure 8:**
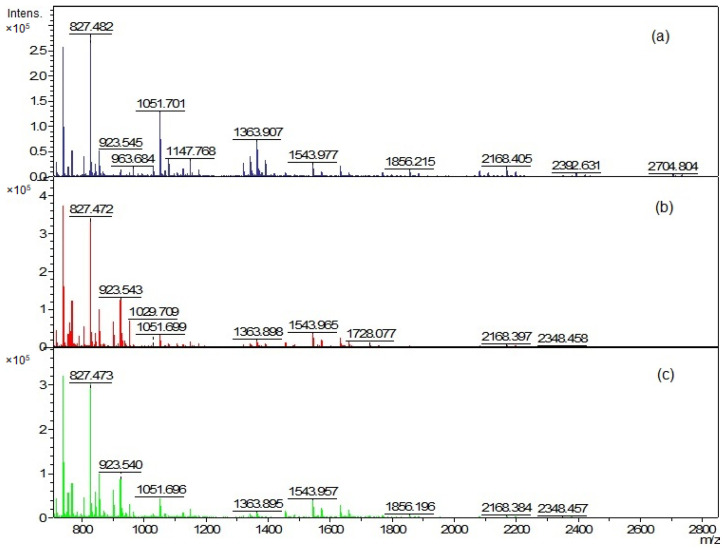
Positive ion MALDI-TOF MS spectra of whole-cell lipid extracts (**a**) *C. acnes* DSM 1897 type IA1, (**b**) *C. acnes* DSM 16379 type IB, and (**c**) *C. acnes* PCM 2334 type II.

MALDI-TOF analysis of cellular lipids was performed, and again similar but distinct mass spectra of different species were obtained (Figure 8). The analysis of the peak list for individual phylotypes (Tables S4–S6) allowed us to confirm the observations previously visible in the TLC analysis: cellular lipid extracts were more abundant in lipids compared to vesicular extracts.

**Figure 9:**
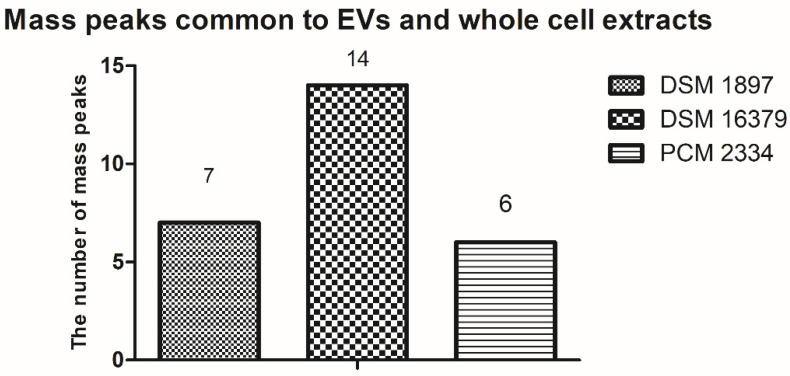
Comparison of the presence of common mass peaks found in EVs and whole-cell lipid extracts of *C. acnes* strains in MALDI-TOF MS analysis.

By comparing the peak list of the EVs and whole-cell extract for the *C. acnes* PCM 2334 strain, the presence of six common mass peaks has been found (717.5 *m*/*z*, 739.4 *m*/*z*, 755.4 *m*/*z*, 827.4 *m*/*z*, 1023.5 *m*/*z*, 1363.8 *m*/*z*) (Tables S3 and S6, Figure 9). After removing from the analysis peaks from the matrix and those that were contaminants from the culture medium (Tables S7 and S8), the number of peaks in the vesicle extract was 30, while there were 76 in the whole-cell lipid extract. *C. acnes* DSM 16379 strain had 25 peaks in its vesicular extract, while the whole-cell extract had 73 peaks; fourteen common mass peaks were found (739.4 *m*/*z*, 755.4 *m*/*z*, 767.5 *m*/*z*, 805.4 *m*/*z*, 827.3 *m*/*z*, 855.5 *m*/*z*, 871.4 *m*/*z*, 901.5 *m*/*z*, 923.5 *m*/*z*, 929.5 *m*/*z*, 951.5 *m*/*z*, 1341.8 *m*/*z*, 1363.8 *m*/*z*, 1391.9 *m*/*z*) (Tables S2 and S5, Figure 9). For the *C. acnes* DSM 1897 strain, the presence of seven common mass peaks was found in whole-cell and vesicular extracts (739.4 *m*/*z*, 755.4 *m*/*z*, 767.5 *m*/*z*, 1341.9 *m*/*z,* 1363.8 *m*/*z,* 2704.7 *m*/*z*, 2732.7 *m*/*z*) (Tables S1 and S4, Figure 9). Both extracts contained 74 and 31 mass peaks, respectively.

## 3. Discussion

The ability of Gram-negative bacteria to produce outer membrane vesicles (OMV) was first observed in the 1960s by Mergenhagen et al. [24]; however, it was long believed that, due to the thick peptidoglycan layer and the lack of an outer membrane, Gram-positive bacteria did not secrete extracellular vesicles. In 1990, conducted research on *Staphylococcus aureus* confirmed the ability of Gram-positive bacteria to release EVs [25]. In these initial studies, vesicle formation was demonstrated using transmission electron microscopy and proteomic analysis [25]. In the group of 90 identified *S. aureus* vesicular proteins, cytoplasmic proteins were the most common, followed by extracellular and membrane proteins. *S. aureus*-derived EVs are especially enriched with extracellular or membrane-associated virulence proteins, including superantigens, hemolysins, coagulation factors, IgG-binding protein, lipase, and others [25]. Next, many further Gram-positive bacterial species were checked to produce EVs; however, compared to Gram-negative bacteria, the information concerning EV content and structures is insufficient. By means of 1D gel separation and LC-MS/MS analysis, 431 proteins in EVs of *C. perfringens* were identified [26]. The most abundant proteins were derived from the membrane or cytoplasm location; also, many bacterial surface proteins were found. These proteins were involved in metabolic processes.

Few studies are focused on the lipid composition of EVs from Gram-positive bacteria. In *Streptococcus pyogenes*, characteristic differences were observed in the contents, distributions, and fatty acid compositions of specific lipids between EVs and bacterial cell membranes [27]. They found phosphatidylglycerol (PG) enrichment and cardiolipin (CL) depletion, a factor known to dictate membrane curvature in EVs. This differential enrichment of EV lipid species relative to the membrane provides evidence for the dedicated mechanism contributing to EV biogenesis.

This is why there is a need to acquire and perform the complex analysis of extracellular vesicles not only from different species but also within different phylotypes belonging to one species. Herein, we performed a comparative analysis of the protein and lipid content of EVs obtained from three *C. acnes* phylotypes representing the same species.

Despite numerous studies on the participation of *C. acnes* in the pathogenesis of acne, its contribution is still unclear. Moreover, subsequent research not only showed the role of this species as the cause of acne but also allowed for the differentiation of the participation of individual phylotypes in various pathological conditions. While the *C. acnes* subsp. *acnes* is usually associated with acne, *C. acnes* subsp. *defendens* is more common in healthy skin and infections of deep tissues [28]. Due to the different properties within individual phylotypes, a comparative analysis is necessary. As the discovery of the release of EVs by Gram-positive bacteria is relatively new, many aspects related to the structure and function remain unknown. Due to the fact that both lipids and proteins are active biomolecules, we decided to perform an initial comparative characterization.

In the following work, thioglycollate-soy broth was used for bacterial culture, due to its beneficial properties for anaerobic microorganisms, instead of the frequently used in EV research brain-heart infusion (BHI) [11,29]. Morphological assessments based on TEM analysis confirmed the presence of closed spherical structures, and the DLS analysis allowed us to determine the size of their diameter in the range of 80.15–93.84 nm, which corresponds to the values previously described [22]; however, Jeon et al. (2017) obtained *C. acnes* EVs with an average diameter of about 38 nm. Research carried out by Jeon et al. [11] confirmed that the EVs proteins differ significantly from individual fractions of cellular proteins. Similar to the strain described therein, in EVs derived from *C. acnes* phylotype II, we found the presence of cytosolic proteins. The sizes of the vesicular proteins obtained from various *C. acnes* strains were different. The EVs obtained from the PCM 2334 strain contained proteins in the range of 37–75 kDa, while the EVs of DSM 1897 and DSM 16379, were 15–75 kDa and 20 kDa, respectively. Protein profiles of bacterial EVs of *Brucella abortus* have also been characterized by SDS-PAGE by Araiza-Villanueva et al. It was shown that the profiles contained proteins in the range of 10–92 kDa for *B. abortus* 2308 and 10–139 kDa for *B. abortus* RB51, which indicates a greater protein richness in this bacterial species [30]. This shows that *C. acnes* EVs seem to have a characteristic narrow protein profile. In proteomic studies of EV of *C. acnes* type I performed by Jeon et al., 252 vesicular proteins were identified, but more than half of them were poorly characterized [11]. Most of the proteins were associated with the cell membrane and with transport, protein processing, translation, and virulence.

In the case of the *C. acnes* EVs lipids analyzed by TLC, there is also a clear differentiation between the phylotypes; it concerns mainly the amount of lipids present in the extracts, from 6 obtained from phylotype IA1, 8 for phylotype II to 11 from phylotype IB. Analysis of the lipidomic profile in *C. acnes* and their EVs have already been performed by Jeon et al. [29]; however, it concerned one strain of the species *C. acnes* (type IA1). Using LC-MS/MS analysis, they identified 214 different lipids in *C. acnes* and EVs, and 187 lipids were shared. *C. acnes* EV contained substantially more PCs, DGs, PAs, PEs, LPAs, LPCs, and MGs than *C. acnes* cells. In our work, which did not use sophisticated methodology like TLC or SDS-PAGE, we were able to show the difference between EVs secreted by different phylotypes.

What is worth mentioning is that the necessary control was also performed to exclude the presence of proteins and lipids derived from the culture medium, which would interfere with the analysis of the samples. It was shown that no contaminants from the culture medium were present in the SDS-PAGE protein fractions and TLC lipid analysis. MALDI-TOF analysis of mass spectra obtained from the medium showed the presence of a few peaks, some of them may be medium-derived contamination and these values were removed from the EV analysis to preserve the reliability of the research.

## 4. Materials and Methods

### 4.1. Bacterial Culture and Isolation of Extracellular Vesicles

The following strains of *Cutibacterium acnes* were used in the studies, namely DSM 1897 (type IA1), whole genome shotgun sequence: AWZZ00000000; DSM 16379 (type IB), whole-genome sequence: AE017283, and PCM 2334 (type II) whole-genome sequence: CP003084.

All *C. acnes* strains were cultivated in thioglycollate-soy broth (TS, Thioglycollate medium, Merck-Millipore, Darmstadt, Germany, Trypticasein soy broth Biomaxima, Lublin, Poland (1:1, *v*/*v*) in anaerobic conditions (GasPack^®^ systems, Becton, Dickinson and Company, Sparks, MD, USA) at 37 °C. Bacterial strains were taken from the TS solid medium with a sterile loop (10 µL) and suspended in 5 mL of the liquid TS medium. After 24 h of incubation, the bacterial suspension was transferred to a culture bottle containing 100 mL of liquid TS medium. After 24 h incubation in anaerobic conditions, 25 mL of the bacterial suspension was transferred into a culture bottle containing 500 mL of liquid TS medium and grown for 48 h. When the culture reached the mid-exponential phase (OD600 = 1–1.5), it was centrifuged (Heraeus Biofuge Stratos, Hanau, Germany) at 4000× *g* for 10 min, and the supernatant was sterilized using a bottle-top 0.22 µm filter (Stericup^®^ Vacuum Filters, Merck-Millipore, Darmstadt, Germany). In the next step, ultrafiltration of the supernatant was carried out using an Amicon^®^ ultrafiltration system, Merck-Millipore, Darmstadt, Germany with 100 kDa Ultrafiltration Discs, Merck-Millipore, Darmstadt, Germany. Then, the concentrate was centrifuged at 4000× *g* (Heraeus Biofuge Stratos, Hanau, Germany) for 15 min at 4 °C, and the obtained supernatant was collected and subjected to another centrifugation (15,000× *g*, 15 min, 4 °C, Hermle Z 36 HK, Wehingen, Germany) followed by ultracentrifugation (100,000× *g*, 2 h, 4 °C, Sorvall WX+, Thermo Fisher, Waltham, MA, USA). The pellet of bacterial EVs obtained was suspended in 1–1.5 mL of the PBS buffer and subjected to further analyses.

To check if the culture medium (TS) did not contain extracellular vesicles, the same volume and the same procedure used to isolate extracellular vesicles were used (i.e., ultrafiltration, differential centrifugation, and ultracentrifugation). The sediment formed after ultracentrifugation and supernatant was used to control SDS-PAGE analysis. Also, the supernatant after ultracentrifugation and Bligh-Dyer extraction was used as a control in TLC and MALDI-TOF MS analyses.

### 4.2. TEM and DLS

EVs were analyzed using transmission electron microscopy (TEM). For TEM analysis, 5 µL of the EVs sample in PBS was loaded on a 400 mesh copper grid with formvar/carbon film and left to absorb for 60 s. Then, the preparation was dried with filter paper. Next, it was negatively stained with 2% uranyl acetate. The images were taken using a JEM-F200 transmission electron microscope (JEOL, Akishima, Japan).

Measurements of the size of bacterial EVs were carried out using dynamic light scattering analysis (DLS, Malvern Zetasizer, Malvern, UK). Extracellular vesicles dissolved in PBS buffer (100 µL) were measured in triplicate on DLS Malvern Zetasizer Nano ZS using Zetasizer software version 8.01.4906.

### 4.3. SDS-PAGE Analysis of EV and Cutibacterium Protein Fractions

The obtained EVs were analyzed with SDS-PAGE together with protein fractions derived from individual *C. acnes* strains. To prepare protein fractions, the culture of *C. acnes* was centrifuged, Heraeus Biofuge Stratos, Hanau, Germany (4000× *g*, 10 min, 4 °C) and washed three times with PBS. The bacterial pellet obtained in this way was re-suspended in PBS and divided into four parts.

The first one, which was dedicated to obtaining bacterial surface proteins, was centrifuged again, Heraeus Biofuge Stratos, Hanau, Germany (4000× *g*, 10 min, 4 °C), suspended in freshly prepared 1 M lithium chloride solution (3 mL for 1 g of the bacterial pellet), and shaken for 30 min at room temperature. Then, it was centrifuged, Heraeus Biofuge Stratos, Hanau, Germany (4000× *g*, 10 min, 4 °C), and the supernatant was collected and precipitated with cold ethanol in a ratio of 1:9 (protein fraction—ethanol 96%, *v*/*v*) and left overnight at 4 °C. After overnight precipitation, the proteins were centrifuged (4000× *g*, 10 min, 4 °C), suspended in 2 mL of MQ water, and dialyzed for 72 h (Dialysis membrane ZelluTrans/ROTH, MWCO 6000–8000, Carl Roth GmbH + Co., Karlsruhe, Germany). The concentration of the surface proteins fraction and EVs fraction was determined using the bicinchoninic acid (BCA) protein assay (Pierce™ BCA Protein Assay Kit, Thermo Fisher, Rockford, IL, USA).

Further protein fractions, i.e., cell wall, cytosol, and whole-cell lysate, were prepared according to Jeon et al. [11]: the cell wall fraction was prepared using lysozyme, whole-cell lysate (using sonication), and cell cytoplasm (using sonication and gradual centrifugation). Bacterial EVs, together with corresponding protein fractions, were analyzed at a concentration of 10 µg per well on SDS-PAGE (4% polyacrylamide stacking gel, 12.5% polyacrylamide separating gel). Proteins were detected by silver staining.

### 4.4. Lipid Extraction

For lipid analysis, *C. acnes* strains (DSM 16379, DSM 1897, and PCM 2334) were cultivated in thioglycollate-soy broth (TS), as above, in an anaerobic chamber (using GasPack systems, Becton, Dickinson and Company, Sparks, MD, USA) at 37 °C for 5 days to reach the stationary growth phase. The culture was then centrifuged at 4000× *g*, for 10 min, and washed twice with PBS and once with MQ water. The pellet obtained was deep-frozen at −80 °C and subsequently freeze-dried. Both EVs and dry cell mass of *C. acnes* were extracted according to the Bligh-Dyer method to obtain lipids [31]. For lipid analysis, 50 mg of dry cell mass and the 0.5 milliliters of EVs fraction and as control, 0.5 mL of TS medium were used. Dry cell mass was suspended in 1 mL of MQ water and then 3.75 mL (1.875 mL for EVs and TS medium fractions) of methanol-chloroform (2:1, *v*/*v*) was added. The mixtures were shaken for 24 h at 20 °C. After centrifugation, the extract was decanted into another tube and the residue was re-suspended in 4.75 mL (2.375 mL for EVs and TS medium fractions) of methanol-chloroform-water (2:1:0.8, *v*/*v*/*v*) and shaken for 2 h at 20 °C. After centrifugation, the supernatant extracts were combined and 2.5 mL (1.25 mL for EVs and TS medium fractions) each of chloroform and MQ water were added. After shaking for 3 min, the mixture was centrifuged to separate the water and chloroform phases completely. The chloroform phase containing crude lipids was withdrawn, brought to dryness under a stream of nitrogen, and stored at −20 °C.

### 4.5. TLC Analysis

Lipids were dissolved in chloroform-methanol (2:1, *v*/*v*) and applied at a concentration of 50 mg/mL to a 10 cm × 10 cm HPTLC Silica gel 60 (Merck-Millipore, Darmstadt, Germany). The TLC plate was developed in the first direction using solvent system (I) chloroform-methanol-water (65:25:4, *v*/*v*) for 25 min at 20 °C, and then after drying, in the second direction with the solvent system (II) chloroform-methanol-acetic acid-water (80:15:12:4, *v*/*v*) under the same conditions. After evaporation of the mobile phase, the plate was sprayed with a reagent containing 300 mM CuSO_4_ in 8.5% phosphoric acid [32] and then placed at 130 °C for 20 min to visualize all lipids.

### 4.6. MALDI-TOF MS Analysis

The lipids were dissolved in chloroform-methanol (2:1, *v*/*v*) and applied to the target plate (MTP 384 ground steel BC, Bruker, Billerica, MA, USA) at a concentration of 1 mg/mL. The matrix used was norharmane at a concentration of 10 mg/mL in the same solvent. We used the dried droplet preparation protocol, 1 µL of the sample after drying on air was overlaid with 1 µL of the matrix solution. The MALDI-TOF analysis was performed with the Ultraflextreme mass spectrometer (Bruker Daltonics, Bremen, Germany) in positive ion mode using FlexAnalysis software (version 3.4). The spectrometer was calibrated using peptide calibration standard II (Bruker Daltonics, Bremen, Germany) with a mass range of 757.399 to 3147.471 *m*/*z*.

## 5. Conclusions

In this work, for the first time, we conducted a comparative analysis of the protein and lipid profiles of EVs obtained from three different *C. acnes* phylotypes. The applied techniques allowed us to show that bacterial EVs differ significantly in protein and lipid composition, depending on the phylotype from which they are isolated. This requires an individual approach to studying these nanostructures within a species. Our results are the background for further analyses that will allow the precise characterization of the qualitative protein and lipid composition. Due to our in-depth understanding of the structure and biological functions of bacterial EVs, they could become a useful tool as drug carriers or in vaccine production.

## Data Availability

The data used to support the findings of this study are included within the article and Supplementary Materials.

## Supplementary Materials

The following supporting information can be downloaded at: https://www.mdpi.com/article/10.3390/ijms23105797/s1.
