# Supplementary material for: Different Cutibacterium acnes Phylotypes Release Distinct Extracellular Vesicles
Source: Int J Mol Sci. 2022 May 21;23(10):5797. doi: 10.3390/ijms23105797 (PMC9147970; doi:10.3390/ijms23105797)
Supplement: Supplementary file 1 [file ijms-23-05797-s001.zip › ijms-1685263-supplementary.pdf]

Supplementary material

Different *Cutibacterium acnes* phylotypes release distinct extracellular vesicles

Anna Chudzik, Paweł Migdał, Mariola Paściak

SDS-PAGE analysis of pure culture medium in order to exclude the influence of the medium on the measurement (Fig. S1). The absence of proteins in lanes 2-4 indicates the absence of media derived proteins by SDS-PAGE analysis.

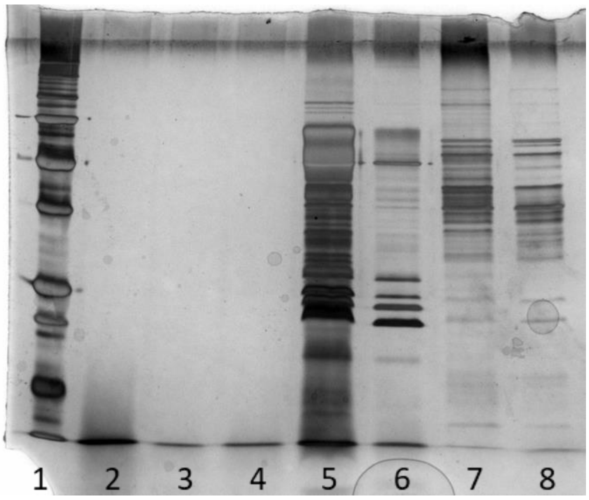

Figure S1. SDS-PAGE of pure culture medium and EVs fractions in order to exclude the influence of the medium on the measurement. 1. Marker. 2. Pure culture medium sterilized using filters with a 0.22 µm membrane. 3. The sediment formed after ultracentrifugation and the procedure identical to that during the extraction of EVs was carried out. 4. Supernatant formed after ultracentrifugation and the procedure identical to that during the extraction of EVs was carried out. 5-6. EVs isolated from *C. acnes* DSM 1897 (different concentrations), 7-8. EVs isolated from *C. acnes* PCM 2334 (different concentrations).

TLC lipid profile analysis of pure culture medium in order to exclude the influence of the medium on the measurement (Fig. S2). The absence of lipid spots proves the absence of lipids in the medium obtained after ultracentrifugation and the procedure identical to that in the collection of EVs.

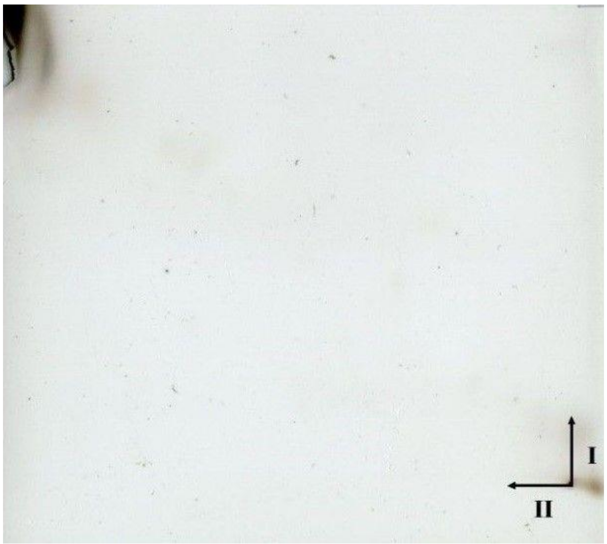

Figure S2. 2D-TLC lipid analysis of medium obtained after ultracentrifugation and the procedure identical to that in the collection of EVs. The TLC was developed in (I) chloroform-methanol-water (65:25:4, v/v), II chloroform-methanol-acetic acid-water (80:15:12:4, v/v) and for the detection of lipids copper sulphate reagent and heating at 130 °C were applied.

List of MALDI-TOF MS mass peaks for EVs lipid extract of *C. acnes* strains

Table S1. List of MALDI-TOF MS mass peaks for EVs lipid extract of *C. acnes* DSM 1897 strain. Yellow lines have been removed from the analysis as medium contamination

m/z	time	Intens.	SN	Quality Fac.	Res.	Area	Rel. Intens.	FWHM	Chi^2
711,389	42172,89	25563,309	34,636	36116,541	8015,775	4003,673	0,125	0,089	206717,160
733,367	42813,17	18993,222	25,610	15318,980	7095,269	3544,881	0,093	0,103	278931,075
739,419	42987,78	19890,160	26,771	17291,731	7822,527	3381,346	0,097	0,095	244135,607
755,390	43445,15	7731,539	9,954	2209,243	7118,319	1451,965	0,038	0,106	118491,887
767,450	43787,31	6946,941	8,782	651,892	6328,500	1423,414	0,034	0,121	338525,905
821,036	45275,92	5795,718	6,920	2039,308	2289,473	3808,923	0,028	0,359	172808,985
827,431	45450,28	10663,669	13,286	6118,079	7884,772	2014,940	0,052	0,105	91629,531
843,391	45882,47	9055,039	11,160	1161,762	6532,557	2111,055	0,044	0,129	484817,382
860,390	46338,33	30430,704	38,961	50135,102	8486,466	5879,593	0,148	0,101	322173,795
865,419	46472,33	34006,188	43,538	46918,562	8170,623	6848,301	0,166	0,106	578305,111
867,443	46526,14	11709,005	14,524	6688,120	6615,598	3019,078	0,057	0,131	140784,949
882,375	46921,24	32232,199	41,083	21524,687	7309,481	7696,830	0,157	0,121	1297085,461
889,454	47107,36	7477,423	8,903	6149,865	8585,773	1391,908	0,036	0,104	39477,889
898,335	47339,82	5697,298	6,582	1023,760	5085,481	1991,501	0,028	0,177	195052,823
911,424	47680,33	205162,012	263,557	82657,014	7745,196	48505,837	1,000	0,118	28028602,253
927,401	48092,68	30801,950	38,671	60177,262	8662,161	6593,274	0,150	0,107	325134,240
933,409	48246,81	145036,643	185,793	97633,825	7676,709	35976,628	0,707	0,122	12163930,681
949,385	48654,28	29394,690	36,726	18891,211	8181,867	6972,449	0,143	0,116	1080184,750
982,474	49487,38	10484,347	12,537	3876,504	8253,082	2412,480	0,051	0,119	152821,970
987,512	49612,97	13059,084	15,859	5597,823	9200,691	2740,584	0,064	0,107	232135,225
998,457	49884,74	6912,082	7,985	1569,541	8413,088	1620,641	0,034	0,119	163599,592
1010,497	50181,97	8088,028	9,518	331,259	9599,011	1652,112	0,039	0,105	1177432,862
1024,507	50525,62	13383,308	16,245	5431,398	8792,507	3071,956	0,065	0,117	198178,006
1033,497	50744,88	14192,704	17,201	1942,174	8744,182	3435,751	0,069	0,118	1084420,200
1046,492	51060,15	9055,760	10,657	888,774	7865,347	2404,554	0,044	0,133	550209,183
1076,518	51781,16	13080,114	15,809	4041,520	9523,034	3106,848	0,064	0,113	509666,890
1078,530	51829,11	12007,463	14,405	1079,739	8143,272	3413,122	0,059	0,132	1071275,215
1100,548	52350,95	7969,839	9,258	826,002	10176,066	1688,577	0,039	0,108	473422,382
1105,501	52467,62	12527,914	15,060	9673,942	9512,755	3157,582	0,061	0,116	168424,821
1108,585	52540,14	9193,187	10,746	922,335	5403,019	4025,552	0,045	0,205	743482,562
1122,523	52866,58	147330,594	187,663	83908,915	8566,182	43616,031	0,718	0,131	15014771,209
1138,500	53238,29	21368,570	26,324	29112,798	8945,204	6216,232	0,104	0,127	342254,531
1144,511	53377,46	179717,319	229,447	119147,364	7561,536	63955,645	0,876	0,151	19284575,954
1160,491	53745,67	32942,944	41,399	30252,573	8162,106	11022,490	0,161	0,142	1300558,578
1193,572	54499,92	5597,985	6,303	1358,509	10367,845	1327,398	0,027	0,115	122045,955
1209,562	54860,74	5830,978	6,672	1002,305	8106,156	1952,483	0,028	0,149	226131,207
1222,604	55153,25	6577,621	7,695	262,852	8316,601	2122,177	0,032	0,147	1124036,900
1231,554	55353,08	6342,114	7,415	1111,910	7298,592	2457,971	0,031	0,169	250107,779
1235,616	55443,55	11869,177	14,724	3612,341	9609,657	3482,417	0,058	0,129	326569,666

1244,593	55642,93	8558,530	10,387	882,987	8170,669	3057,712	0,042	0,152	591163,953
1257,599	55930,51	13484,477	16,936	4451,135	8956,620	4550,149	0,066	0,140	566959,339
1341,852	57758,47	8036,773	10,179	2773,941	10301,067	2514,128	0,039	0,130	156031,703
1344,642	57818,02	5130,420	6,193	645,612	7030,599	2351,539	0,025	0,191	329457,326
1363,818	58225,51	5756,157	7,177	664,707	3619,862	4809,256	0,028	0,377	569999,877
1385,647	58685,93	25508,710	35,657	82244,602	10566,960	8928,400	0,124	0,131	302836,479
1401,638	59020,89	5038,231	6,366	815,594	9028,252	1927,789	0,025	0,155	243286,146
1655,818	64108,26	3756,328	6,205	482,298	10446,820	1640,695	0,018	0,158	271538,515
1854,876	67823,80	3492,292	7,602	863,730	11474,875	1813,453	0,017	0,162	147770,805
1985,917	70161,38	12353,784	39,547	39982,662	14517,393	5893,837	0,060	0,137	249924,178
1994,049	70303,86	3025,735	8,624	3663,019	12077,856	1443,714	0,015	0,165	26241,513
2002,911	70458,79	3944,856	11,987	1173,667	12521,488	2076,006	0,019	0,160	164611,974
2090,095	71965,08	2377,494	7,752	1789,172	14364,798	960,937	0,012	0,146	29642,993
2093,129	72016,92	5345,026	20,018	19340,146	14621,301	2500,834	0,026	0,143	41841,817
2207,168	73939,03	1949,459	7,806	2208,915	13029,945	966,667	0,010	0,169	18115,060
2704,701	81793,28	1236,879	10,856	2001,862	9779,409	1078,183	0,006	0,277	13293,905
2732,736	82213,31	1281,717	11,842	2029,625	8387,747	1334,462	0,006	0,326	16224,513

Table S2. List of MALDI-TOF MS mass peaks for EVs lipid extract of *C. acnes* DSM 16379 strain. Green and yellow lines have been removed from the analysis as matrix and medium contamination respectively.

m/z	time	Intens.	SN	Quality Fac.	Res.	Area	Rel. Intens.	FWHM	Chi^2
707,338	42053,80	8595,439	7,516	919,683	4441,440	2291,304	0,061	0,159	414354,978
739,424	42987,91	28358,327	26,694	12987,415	5966,886	6438,395	0,202	0,124	1018176,438
751,356	43330,08	7370,162	6,145	136,931	5559,242	1635,198	0,053	0,135	2008958,273
755,395	43445,30	7266,115	6,033	1232,385	7100,977	1211,827	0,052	0,106	164706,531
767,448	43787,24	8040,943	6,751	255,711	3400,850	2859,946	0,057	0,226	1368277,586
805,455	44848,26	8377,030	7,034	2768,136	7294,448	1521,239	0,060	0,110	93572,530
812,361	45038,32	21036,329	19,403	4735,415	6665,582	4786,721	0,150	0,122	1093730,245
827,431	45450,28	51352,525	48,963	38425,522	6545,634	12634,918	0,366	0,126	2017743,200
834,339	45637,85	20811,809	19,132	8280,880	6277,632	5271,830	0,148	0,133	668657,792
843,401	45882,75	18017,022	16,402	7340,204	6304,412	4455,778	0,128	0,134	542765,575
855,463	46206,68	21401,762	19,694	7632,420	6208,602	5553,900	0,153	0,138	867100,967
860,395	46338,47	25275,696	23,506	22578,781	7131,186	5811,319	0,180	0,121	390119,364
865,419	46472,32	23916,879	22,196	14672,943	6948,444	5585,757	0,171	0,125	477444,820
871,438	46632,16	9504,469	8,077	1455,180	6504,603	2256,629	0,068	0,134	270091,112
882,374	46921,21	38699,333	36,765	21676,120	6714,925	10125,327	0,276	0,131	1802530,738
901,511	47422,68	10958,107	9,411	3232,242	7352,917	2382,519	0,078	0,123	159468,579
911,426	47680,39	136080,805	133,387	89357,489	7450,162	33971,650	0,970	0,122	7853789,461
923,492	47992,14	18577,521	16,903	6053,450	6951,842	4609,151	0,132	0,133	441541,648
927,404	48092,77	14888,876	13,270	6296,817	6877,195	3822,156	0,106	0,135	281401,703
929,537	48147,54	7654,175	6,068	693,862	6474,534	1856,594	0,055	0,144	335918,333
933,405	48246,72	140238,087	137,627	95359,211	7354,664	36901,130	1,000	0,127	10424579,602
949,375	48654,02	21313,840	19,712	3493,346	6902,048	5983,258	0,152	0,138	1104404,671
951,536	48708,87	11902,998	10,330	591,907	4132,780	4875,807	0,085	0,230	1409719,889
982,482	49487,58	9171,360	7,741	2035,378	7716,328	2022,233	0,065	0,127	175255,265
987,507	49612,85	10997,001	9,661	2162,847	7413,730	2657,927	0,078	0,133	260871,764
989,487	49662,13	17611,438	16,361	11232,941	6941,857	4997,039	0,126	0,143	383050,316
1005,459	50057,81	7434,611	6,081	582,390	6973,446	1911,469	0,053	0,144	457798,321
1023,482	50500,55	10847,698	9,661	328,289	7043,958	2989,273	0,077	0,145	2068270,485
1033,491	50744,75	10224,827	9,072	1010,988	7968,762	2530,229	0,073	0,130	517663,068
1045,457	51035,12	13589,235	12,607	536,356	6611,229	4327,770	0,097	0,158	2293019,205
1105,508	52467,78	9662,485	8,743	801,065	7638,958	2779,040	0,069	0,145	741161,843
1122,530	52866,75	48430,864	50,763	76169,818	8252,554	14869,834	0,345	0,136	1213052,237
1144,508	53377,41	74715,953	80,056	47337,910	7885,700	25436,003	0,533	0,145	5960745,112
1160,480	53745,42	11012,760	10,471	1479,603	7266,626	3810,523	0,079	0,160	481557,018
1213,698	54953,67	14810,618	15,330	2410,674	7607,498	5210,922	0,106	0,160	676860,806
1257,601	55930,57	8298,134	8,266	1310,422	6657,697	3372,072	0,059	0,189	329167,473
1341,855	57758,55	6793,626	7,112	1615,698	8160,805	2307,190	0,048	0,164	158891,148
1363,837	58225,92	10362,894	12,220	2339,077	7657,981	4331,814	0,074	0,178	321548,490
1385,649	58685,97	5992,834	6,395	1597,534	8788,259	2014,422	0,043	0,158	124712,257
1391,873	58816,57	6754,319	7,521	612,535	5989,723	3352,738	0,048	0,232	475628,718

1437,921	59773,87	10617,966	13,987	10090,757	10037,082	3697,805	0,076	0,143	133079,341
1465,950	60349,07	7000,917	8,878	1784,702	8261,204	2923,120	0,050	0,177	176414,961

Table S3. List of MALDI-TOF MS mass peaks for EVs lipid extract of *C. acnes* PCM 2334 strain. Yellow lines have been removed from the analysis as medium contamination.

m/z	time	Intens.	SN	Quality Fac.	Res.	Area	Rel. Intens.	FWHM	Chi^2
711,393	42173,01	25249,087	20,923	29540,956	7745,320	4044,495	0,073	0,092	149076,751
717,453	42350,54	8939,198	7,015	3632,921	7746,894	1388,920	0,026	0,093	103718,974
733,370	42813,26	19925,299	16,190	20241,996	6666,730	3908,831	0,058	0,110	150896,112
739,425	42987,95	39700,838	32,918	23511,336	7490,231	7098,064	0,115	0,099	876549,036
755,400	43445,44	13491,777	10,713	3885,651	7095,387	2517,013	0,039	0,106	200225,057
812,365	45038,43	27064,829	21,595	7490,707	7706,214	5306,403	0,078	0,105	1120210,424
827,436	45450,41	25356,072	19,865	11629,126	7322,188	5361,726	0,073	0,113	691243,575
834,345	45638,01	19857,146	15,336	3585,534	6761,357	4675,293	0,057	0,123	1038037,317
843,404	45882,83	16878,082	12,862	1999,943	6244,348	4128,087	0,049	0,135	944631,563
860,397	46338,53	45410,612	35,644	52986,345	7888,167	9428,012	0,131	0,109	741564,875
865,424	46472,46	51969,095	40,835	65880,229	7743,514	11085,086	0,150	0,112	973286,514
867,440	46526,04	17810,212	13,481	3696,974	6918,970	4342,056	0,051	0,125	444557,840
882,380	46921,36	55358,125	43,221	25835,283	7051,983	13723,599	0,160	0,125	3274902,108
889,463	47107,59	14248,120	10,457	9349,387	7949,680	2883,579	0,041	0,112	101955,831
898,348	47340,17	10537,159	7,491	560,704	5571,714	3314,777	0,030	0,161	1351479,924
911,432	47680,55	346406,000	273,331	128858,599	7603,979	83050,036	1,000	0,120	49422099,823
927,410	48092,91	48571,566	37,209	68600,952	8123,200	11030,142	0,140	0,114	773276,102
933,415	48246,98	255667,373	200,702	93687,261	7311,179	66612,652	0,738	0,128	39465583,533
949,394	48654,51	53010,019	40,471	29496,587	7871,341	12937,263	0,153	0,121	2493729,895
982,484	49487,64	17692,619	12,806	4709,504	7911,090	4158,091	0,051	0,124	350569,625
987,520	49613,18	18687,768	13,658	4465,944	8483,287	4198,663	0,054	0,116	507733,013
998,464	49884,92	10394,521	7,137	535,731	7174,517	2805,487	0,030	0,139	1076494,981
1004,468	50033,36	10065,378	6,886	387,631	7393,477	2553,967	0,029	0,136	1529936,470
1008,495	50132,69	17894,414	12,955	1307,475	7656,529	4673,725	0,052	0,132	1912261,045
1010,510	50182,31	12663,105	8,880	334,536	7817,285	3133,250	0,037	0,129	2975472,076
1023,469	50500,25	17280,200	12,455	1124,019	9080,865	3818,280	0,050	0,113	1512018,931
1024,505	50525,57	19606,963	14,275	1672,144	7505,330	5205,536	0,057	0,137	1691982,887
1030,468	50671,11	12761,220	8,903	376,499	6840,665	3930,669	0,037	0,151	2849727,067
1033,504	50745,05	21933,808	16,041	1487,688	7770,787	5970,036	0,063	0,133	3244777,880
1045,466	51035,34	22306,576	16,274	2894,410	6756,692	7140,279	0,064	0,155	2159704,085
1046,507	51060,52	17968,914	12,886	478,105	8863,199	4255,890	0,052	0,118	3686312,869
1061,493	51421,65	10677,721	7,221	302,325	5704,612	3845,160	0,031	0,186	2314922,856
1076,529	51781,43	18155,286	12,998	2653,021	8626,052	4573,592	0,052	0,125	931776,099
1078,538	51829,31	17793,167	12,682	1171,725	8303,395	4730,472	0,051	0,130	1774452,712
1100,563	52351,32	12708,069	8,752	656,674	8858,034	2982,809	0,037	0,124	1624605,559
1105,517	52467,99	23192,673	16,824	14503,199	8490,921	6462,773	0,067	0,130	462159,387
1108,595	52540,36	12988,520	8,894	799,005	5025,601	5939,203	0,037	0,221	1581579,271
1122,537	52866,90	212886,146	163,586	119581,867	8235,001	64496,715	0,615	0,136	19196390,540
1138,517	53238,69	30693,263	22,622	20577,633	8385,404	9160,831	0,089	0,136	791372,651
1144,521	53377,71	289919,776	223,766	56785,526	7273,728	105887,213	0,837	0,157	76850945,618
1160,502	53745,93	54416,615	41,249	28014,219	7833,143	18689,168	0,157	0,148	3249197,938

1209,562	54860,73	9395,384	6,329	486,990	7446,066	3326,044	0,027	0,162	1232459,356
1215,568	54995,64	9008,921	6,048	628,450	7351,377	3182,917	0,026	0,165	831981,459
1222,619	55153,59	9961,933	6,835	243,846	7886,894	3194,175	0,029	0,155	2544194,623
1231,562	55353,26	10268,799	7,107	949,390	7066,188	3977,305	0,030	0,174	735543,629
1235,633	55443,94	17141,165	12,636	2562,311	8889,168	5183,465	0,049	0,139	754150,793
1244,607	55643,24	15196,288	11,165	361,303	7900,964	5457,801	0,044	0,158	4554053,125
1257,612	55930,80	21817,000	16,630	3615,856	8659,444	7372,888	0,063	0,145	1378769,302
1341,876	57758,99	9059,433	6,579	456,859	5931,177	4243,006	0,026	0,226	1386544,798
1363,793	58224,99	9082,693	6,702	1149,793	3496,449	7954,794	0,026	0,390	848232,291
1385,663	58686,26	46040,032	39,231	78578,054	9802,566	16848,314	0,133	0,141	994824,119
1401,655	59021,24	10034,921	7,813	1407,637	8750,518	3893,089	0,029	0,160	546950,509
1655,825	64108,38	5979,858	6,103	954,976	9072,097	2935,693	0,017	0,183	329771,511
1683,843	64644,41	5709,634	6,056	1950,664	9839,222	2711,857	0,016	0,171	139274,494
1854,894	67824,12	5025,469	6,888	1198,024	10679,318	2670,366	0,015	0,174	208425,534
1985,938	70161,75	15044,484	32,700	17361,432	12430,881	8015,668	0,043	0,160	381050,400
2001,921	70441,51	4720,807	9,369	580,936	11498,445	2523,912	0,014	0,174	436550,081
2093,133	72017,00	3254,461	7,295	2353,304	10297,157	1878,581	0,009	0,203	46004,830

List of MALDI-TOF MS mass peaks for cellular lipid extract of *C. acnes* strains

Table S4. List of MALDI-TOF MS mass peaks for cellular lipid extract of *C. acnes* DSM 1897 strain. Yellow lines have been removed from the analysis as medium contamination.

m/z	time	Intens.	SN	Quality Fac.	Res.	Area	Rel. Intens.	FWHM	Chi^2
717,498	42351,87	28512,136	50,122	90435,179	6775,879	5457,589	0,107	0,106	227585,925
723,501	42526,95	4153,259	6,240	468,348	3100,359	1478,959	0,016	0,233	278068,909
739,467	42989,16	244380,862	442,243	128773,644	5516,324	62024,403	0,921	0,134	24417246,417
745,532	43163,41	6055,326	9,401	5168,208	5233,251	1390,901	0,023	0,142	100734,777
753,486	43390,88	6771,023	10,695	5367,739	4913,012	1701,424	0,026	0,153	150574,295
755,445	43446,71	19366,872	33,531	55120,488	5861,055	4547,597	0,073	0,129	210786,670
767,498	43788,66	50091,002	89,859	82059,500	5868,889	12360,575	0,189	0,131	1262950,743
783,467	44237,58	4880,098	7,352	697,299	5215,949	1172,607	0,018	0,150	471267,087
805,509	44849,75	39853,433	71,668	84317,235	7122,287	8534,269	0,150	0,113	602191,515
811,505	45014,80	4659,530	6,864	718,237	4598,969	1291,481	0,018	0,176	304402,356
823,380	45339,93	4385,766	6,059	722,114	2704,925	2109,492	0,017	0,304	181554,007
825,302	45392,32	10944,765	18,247	333,827	4760,807	3521,487	0,041	0,173	20582235,008
827,482	45451,65	265381,924	491,083	134954,999	5493,748	79979,327	1,000	0,151	34073247,574
833,543	45616,28	7229,866	11,359	9660,258	5380,599	1914,839	0,027	0,155	106194,403
839,500	45777,49	6058,775	9,194	4018,164	4546,447	1854,424	0,023	0,185	142304,173
841,506	45831,65	6336,874	9,721	1779,669	4173,993	2119,421	0,024	0,202	425721,210
843,462	45884,39	23275,836	41,356	29101,777	5939,441	6385,084	0,088	0,142	677112,913
855,514	46208,03	46930,806	85,820	85067,632	5737,624	14036,251	0,177	0,149	1530466,371
867,561	46529,26	9119,753	15,006	14053,876	5724,235	2436,437	0,034	0,152	132157,286
871,493	46633,63	5908,694	8,996	7031,210	6046,058	1438,897	0,022	0,144	73003,041
901,570	47424,20	4861,946	7,006	4179,641	5296,155	1328,104	0,018	0,170	56892,022
921,604	47943,49	5002,875	7,415	4637,261	877,677	7199,113	0,019	1,050	71677,937
923,545	47993,51	7875,416	12,723	12955,591	6214,272	2081,523	0,030	0,149	113371,518
952,062	48722,20	5820,610	9,865	2428,919	627,113	13053,889	0,022	1,518	284246,811
963,684	49016,05	15716,251	28,011	28735,733	5765,382	5232,972	0,059	0,167	340495,775
981,625	49466,19	5580,183	8,559	11855,676	6798,902	1345,007	0,021	0,144	29898,076
991,722	49717,69	5061,559	7,599	1967,865	4742,926	1728,599	0,019	0,209	130372,106
1009,652	50161,17	5377,176	8,462	10176,689	6516,282	1446,000	0,020	0,155	38336,701
1029,729	50653,09	18659,786	35,438	125079,140	7997,365	4911,805	0,070	0,129	91992,554
1051,701	51185,98	127013,459	257,122	192479,715	6933,297	43503,553	0,479	0,152	6023529,883
1057,762	51331,97	5427,943	8,788	8764,571	7052,917	1450,610	0,020	0,150	47658,508
1065,717	51522,98	4753,033	7,427	2328,725	5716,893	1551,746	0,018	0,186	115954,911
1067,687	51570,16	11371,161	20,994	28419,395	6872,687	3575,021	0,043	0,155	150094,189
1079,739	51857,91	33172,051	65,911	77898,831	6813,592	11717,560	0,125	0,158	833454,411
1095,717	52236,92	5926,663	9,871	11020,113	7593,226	1596,689	0,022	0,144	51022,422
1105,761	52473,74	7839,069	13,922	1808,693	6436,058	2680,023	0,030	0,172	988805,324
1125,795	52942,92	15228,749	29,373	81470,451	8322,557	4384,257	0,057	0,135	107153,514
1139,681	53265,66	4979,949	7,961	4271,194	5997,853	1763,568	0,019	0,190	82197,083
1147,768	53452,73	30433,057	62,133	90936,929	8300,889	9657,408	0,115	0,138	511773,680
1153,848	53592,92	6925,320	12,172	1907,675	3416,339	4535,435	0,026	0,338	364214,606

1175,800	54096,05	13106,803	25,735	31364,345	7670,947	4466,980	0,049	0,153	233223,297
1319,936	57288,66	25378,918	57,866	38060,813	8702,709	9544,555	0,096	0,152	978695,947
1335,890	57631,04	3886,822	6,628	3354,837	6343,725	1550,037	0,015	0,211	47145,080
1341,923	57760,00	40708,729	95,560	152691,333	8820,879	16043,052	0,153	0,152	766195,540
1347,965	57888,83	11771,145	25,676	14298,828	7238,622	5246,511	0,044	0,186	479145,925
1357,915	58100,38	3864,720	6,504	2593,418	9084,179	1047,353	0,015	0,149	49368,875
1363,907	58227,39	68041,930	163,035	164772,910	7244,634	34782,977	0,256	0,188	2897076,156
1369,962	58355,47	16289,137	36,462	73656,257	8303,886	6708,408	0,061	0,165	211113,121
1379,895	58564,96	7465,616	14,974	9665,852	5898,893	3979,919	0,028	0,234	184850,820
1390,546	58788,74	14222,089	31,693	1283,668	3359,825	12968,542	0,054	0,414	3374068,047
1391,954	58818,25	23205,396	53,822	14591,818	4562,928	18111,776	0,087	0,305	3344245,070
1418,584	59373,77	4679,218	8,608	2587,687	1608,712	8195,680	0,018	0,882	150280,911
1419,983	59402,81	4220,444	7,151	698,021	15708,415	659,792	0,016	0,090	78219,384
1455,959	60144,67	8530,847	18,776	22862,311	7524,225	4154,742	0,032	0,194	148262,829
1484,006	60716,68	3782,651	6,785	752,029	4442,546	2439,387	0,014	0,334	133895,626
1543,977	61921,85	24763,994	63,391	47102,201	8231,701	13587,110	0,093	0,188	1084770,028
1572,036	62477,65	7714,073	17,785	4167,027	5343,682	5610,780	0,029	0,294	643601,472
1631,998	63648,95	20164,212	54,578	63165,100	8579,734	11594,127	0,076	0,190	601917,189
1660,036	64189,24	7037,897	17,111	13209,580	6335,340	5008,161	0,027	0,262	153624,692
1768,207	66231,96	8202,953	22,160	17336,559	6981,751	6297,611	0,031	0,253	238968,441
1796,245	66751,14	4061,210	9,419	3633,058	5999,751	3126,866	0,015	0,299	88458,286
1856,215	67848,10	12003,892	35,627	48832,332	8559,529	8768,632	0,045	0,217	283821,428
1884,254	68354,89	5456,872	15,033	11013,626	6434,445	4759,082	0,021	0,293	99151,894
2080,396	71799,09	10501,021	40,558	35692,228	8389,157	8995,541	0,040	0,248	300224,850
2108,436	72277,92	6043,683	22,499	8367,755	6951,062	5794,005	0,023	0,303	248710,730
2152,429	73022,79	2286,003	6,419	876,029	6567,799	1696,676	0,009	0,328	57422,160
2168,405	73291,39	13939,470	60,475	46728,445	8971,450	11989,752	0,053	0,242	493656,291
2182,430	73526,38	2553,523	7,669	773,067	6354,886	2067,154	0,010	0,343	91470,456
2196,442	73760,41	7960,019	33,890	14532,870	7412,900	7899,584	0,030	0,296	289354,365
2224,462	74226,13	2380,124	7,251	1409,593	5362,946	2228,313	0,009	0,415	46339,972
2392,631	76961,53	4620,625	23,709	11217,028	7703,400	4746,860	0,017	0,311	92596,880
2420,660	77407,94	3291,441	16,110	4430,245	7150,388	3426,010	0,012	0,339	51783,560
2682,817	81463,89	1328,403	6,221	1618,637	8952,617	895,758	0,005	0,300	10293,872
2688,825	81554,45	1281,553	6,190	1547,741	5673,978	1351,277	0,005	0,474	15279,461
2704,804	81794,83	2891,572	20,794	4064,295	7501,219	3553,304	0,011	0,361	51366,531
2732,806	82214,36	2319,023	16,266	3630,970	7026,967	2955,266	0,009	0,389	41288,020

Table S5. List of MALDI-TOF MS mass peaks for cellular lipid extract of *C. acnes* DSM 16379 strain. Yellow lines have been removed from the analysis as medium contamination.

m/z	time	Intens.	SN	Quality Fac.	Res.	Area	Rel. Intens.	FWHM	Chi^2
717,480	42351,32	44956,131	66,753	68937,611	6814,883	8630,517	0,120	0,105	577458,094
732,414	42785,61	6289,750	8,112	755,174	1769,389	3638,656	0,017	0,414	472443,441
739,462	42989,01	373870,197	575,463	115667,532	4829,546	109373,383	1,000	0,153	72921282,313
745,518	43163,01	12047,132	16,759	3023,692	5735,884	2652,768	0,032	0,130	550636,786
753,484	43390,82	31790,875	47,540	33639,623	5617,917	7747,372	0,085	0,134	825461,173
755,438	43446,50	34198,475	51,263	31018,498	5607,786	8499,195	0,091	0,135	1104075,032
761,488	43618,51	65255,890	100,285	71591,558	6240,828	14926,510	0,175	0,122	1765373,637
767,502	43788,78	120151,099	187,228	125938,766	5349,628	32649,157	0,321	0,143	5321875,593
772,764	43937,22	8739,909	11,855	3208,746	2078,674	4950,990	0,023	0,372	185936,585
775,504	44014,29	7925,403	10,549	5085,995	5398,373	1804,783	0,021	0,144	59230,656
777,469	44069,52	6390,319	8,136	578,919	5201,856	1498,889	0,017	0,149	327209,851
783,467	44237,58	10001,408	13,941	2181,849	5282,518	2520,756	0,027	0,148	574812,303
789,521	44406,56	30698,223	46,965	30000,804	6142,722	7305,919	0,082	0,129	824550,592
800,709	44717,15	5073,126	6,165	1195,817	1847,151	2909,073	0,014	0,433	144035,843
805,494	44849,33	55399,575	86,996	65706,168	7015,037	12083,273	0,148	0,115	1240599,331
813,459	45068,47	5600,970	7,076	1871,093	4288,657	1698,850	0,015	0,190	77336,293
819,515	45234,36	5198,181	6,462	312,081	4721,936	1338,548	0,014	0,174	439064,564
827,472	45451,38	330211,478	535,470	113680,686	5124,029	108228,398	0,883	0,161	60620722,300
833,530	45615,93	12722,081	18,574	11348,174	6241,901	3069,309	0,034	0,134	200129,952
839,566	45779,27	6246,628	8,034	401,493	2791,661	2906,154	0,017	0,301	540587,587
841,493	45831,30	26540,143	41,251	17306,490	5376,213	8143,352	0,071	0,157	1314565,702
843,454	45884,18	32906,520	51,879	25922,900	5808,969	9385,620	0,088	0,145	1386700,938
855,509	46207,90	92562,543	150,323	94657,900	5446,170	29616,360	0,248	0,157	5145575,036
867,567	46529,44	6046,694	7,801	472,129	4084,320	2041,229	0,016	0,212	341191,343
871,484	46633,40	9060,617	12,818	3211,720	6100,032	2304,401	0,024	0,143	247300,113
901,558	47423,90	68892,262	113,706	132079,322	7449,732	16749,687	0,184	0,121	1144712,911
915,573	47787,78	10310,418	15,361	7563,111	6919,304	2445,343	0,028	0,132	139104,949
923,543	47993,44	125863,187	211,295	113712,236	6629,707	36418,438	0,337	0,139	6859474,821
929,593	48148,97	36176,091	59,295	118098,604	7195,342	9339,093	0,097	0,129	330248,773
937,561	48353,05	17479,253	27,552	27257,958	6364,064	5051,081	0,047	0,147	269174,698
939,518	48403,03	8325,160	11,985	1095,098	5896,530	2400,873	0,022	0,159	563060,076
951,577	48709,90	68208,143	114,290	160627,118	6348,498	21541,634	0,182	0,150	1696935,024
964,584	49038,73	4892,564	6,197	781,390	3399,837	2243,765	0,013	0,284	174509,776
967,552	49113,46	5150,449	6,645	960,416	6039,937	1353,392	0,014	0,160	105894,477
1029,709	50652,61	6458,316	9,844	3606,994	6937,323	1705,391	0,017	0,148	51995,020
1051,699	51185,92	31237,407	56,573	94837,020	6679,986	10660,010	0,084	0,157	462664,475
1079,732	51857,75	8867,896	14,898	9706,638	6214,081	3119,054	0,024	0,174	116360,107
1105,761	52473,74	9803,955	16,916	8281,251	6044,790	3692,665	0,026	0,183	195355,941
1125,782	52942,62	8069,201	13,595	4675,200	7587,180	2335,054	0,022	0,148	128682,564
1147,770	53452,77	15105,447	28,167	39772,008	7260,481	5212,942	0,040	0,158	159814,158
1153,826	53592,40	4636,859	6,792	699,960	3996,656	2261,553	0,012	0,289	165245,513

1175,799	54096,03	8516,517	15,055	9362,035	6643,455	3139,141	0,023	0,177	115838,330
1319,922	57288,36	6092,970	11,760	2619,863	7010,897	2407,060	0,016	0,188	100528,361
1341,909	57759,69	10401,292	22,248	45177,501	7844,455	4221,089	0,028	0,171	65615,595
1363,898	58227,21	17967,769	41,016	42339,412	6645,926	9565,312	0,048	0,205	372234,334
1369,935	58354,89	5492,829	10,579	4666,627	7285,931	2241,971	0,015	0,188	53476,852
1377,916	58523,28	4756,955	8,833	519,359	5318,576	2583,897	0,013	0,259	316505,855
1390,534	58788,50	4148,214	7,403	446,050	2761,183	3556,846	0,011	0,504	357985,500
1391,945	58818,06	7881,382	16,645	2085,866	4314,374	5896,795	0,021	0,323	355742,056
1455,948	60144,45	14221,556	34,248	39632,281	7308,904	7512,501	0,038	0,199	249076,779
1469,953	60430,76	3541,957	6,185	966,585	6951,078	1417,854	0,009	0,211	67876,184
1477,980	60594,25	3936,700	7,167	1320,700	7217,636	1604,437	0,011	0,205	63490,656
1483,983	60716,22	7686,361	17,292	4032,474	5744,022	4894,588	0,021	0,258	185660,743
1543,965	61921,62	35122,297	95,859	106796,134	7168,500	22420,964	0,094	0,215	1020063,303
1557,983	62199,90	6989,723	16,075	2049,274	6482,500	4179,562	0,019	0,240	347123,344
1566,009	62358,68	4257,354	8,296	1988,557	5224,968	2688,341	0,011	0,300	68258,602
1572,006	62477,05	17418,550	46,266	14127,166	5987,366	13155,711	0,047	0,263	808756,208
1631,988	63648,76	24836,471	71,420	59422,257	7482,484	16563,094	0,066	0,218	778504,150
1640,050	63804,59	4436,353	9,173	1750,957	5240,110	3015,729	0,012	0,313	84515,516
1646,009	63919,52	5418,908	12,239	997,100	6549,911	3230,866	0,014	0,251	235771,622
1660,027	64189,07	12180,790	33,481	12119,154	6251,162	9436,992	0,033	0,266	335432,822
1668,081	64343,42	3753,263	7,194	1331,024	4884,056	2603,081	0,010	0,342	77675,673
1728,077	65481,66	4986,155	11,943	2967,459	5486,032	3900,105	0,013	0,315	82808,902
1756,099	66006,49	4435,589	10,358	5596,184	5421,786	3547,518	0,012	0,324	38548,003
1768,207	66231,95	3095,643	6,460	1446,543	3879,926	2973,328	0,008	0,456	66733,568
1856,205	67847,92	3506,354	8,889	3957,539	7001,367	2380,287	0,009	0,265	27822,991
2080,381	71798,84	3662,186	13,530	4212,150	7384,606	3016,324	0,010	0,282	41774,955
2108,410	72277,48	2868,921	10,191	3103,326	6532,807	2508,003	0,008	0,323	33281,785
2168,397	73291,27	4575,567	19,952	4634,208	7524,259	4139,364	0,012	0,288	70532,095
2182,417	73526,16	1985,049	6,489	1250,809	6110,613	1613,944	0,005	0,357	32238,370
2196,428	73760,16	3731,660	16,209	3377,392	6738,427	3671,155	0,010	0,326	64316,171
2348,458	76252,61	2158,972	9,202	2876,290	6429,066	1985,565	0,006	0,365	20731,626
2376,485	76703,18	1768,352	7,169	1845,376	5187,510	1819,107	0,005	0,458	21764,043

Table S6. List of MALDI-TOF MS mass peaks for cellular lipid extract of *C. acnes* PCM 2334 strain. Green and yellow lines have been removed from the analysis as matrix and medium contamination.

m/z	time	Intens.	SN	Quality Fac.	Res.	Area	Rel. Intens.	FWHM	Chi^2
707,369	42054,71	16490,297	27,874	5718,758	4683,360	4501,008	0,054	0,151	700325,247
711,433	42174,20	8796,179	14,160	3805,666	5113,000	2042,784	0,029	0,139	93561,264
717,487	42351,54	43315,366	76,486	41680,400	6734,846	8363,335	0,141	0,107	635608,698
732,399	42785,18	4391,509	6,038	886,659	1292,176	3239,320	0,014	0,567	181517,794
735,396	42871,77	5149,537	7,446	614,048	4895,417	1190,936	0,017	0,150	172650,564
739,459	42988,93	307217,078	563,622	76404,332	5263,389	81983,488	1,000	0,140	61085101,550
745,519	43163,04	9654,535	15,563	3806,004	6637,395	1775,796	0,031	0,112	97824,901
751,399	43331,32	12218,052	20,317	3379,280	5705,612	2783,961	0,040	0,132	360449,679
753,474	43390,54	11846,563	19,648	999,546	5897,182	2619,649	0,039	0,128	1016986,766
755,435	43446,43	53392,897	96,913	38347,019	6110,405	12374,620	0,174	0,124	1882443,672
761,488	43618,49	5230,411	7,449	1236,954	6345,887	913,545	0,017	0,120	68298,866
767,492	43788,48	74325,909	136,811	77728,879	5980,765	18059,878	0,242	0,128	2492556,620
772,771	43937,40	6233,569	9,390	974,279	1499,126	4617,306	0,020	0,515	351182,341
783,462	44237,45	12313,898	20,937	4757,835	5984,131	2840,860	0,040	0,131	247057,701
795,422	44570,66	9944,045	16,675	1804,966	4195,208	3340,899	0,032	0,190	287412,336
799,436	44681,92	5348,073	7,953	213,833	5260,045	1254,368	0,017	0,152	550296,344
805,497	44849,40	45891,597	85,788	37383,672	7464,882	9373,095	0,149	0,108	962464,290
825,303	45392,33	5323,769	7,720	7,029	3431,663	2035,070	0,017	0,240	27982770,589
827,473	45451,42	293091,232	566,747	88689,881	5488,939	88517,045	0,954	0,151	55174813,596
833,532	45615,99	13940,815	24,541	15750,801	6752,244	3100,481	0,045	0,123	107590,082
839,456	45776,32	7175,856	11,375	1206,412	3929,918	2562,436	0,023	0,214	214030,404
841,490	45831,21	12246,955	21,305	1110,182	5208,259	3591,380	0,040	0,162	1626341,920
843,450	45884,08	55878,298	106,484	38782,892	6247,641	14950,262	0,182	0,135	2648660,704
855,507	46207,84	95108,577	184,182	92380,352	6065,579	27073,672	0,310	0,141	4471989,067
859,464	46313,62	5096,313	7,380	1004,239	2883,166	2286,073	0,017	0,298	128752,642
867,537	46528,62	4873,809	6,971	1147,878	3652,324	1696,642	0,016	0,238	84310,665
871,481	46633,30	17305,373	31,473	12472,579	6315,694	4582,768	0,056	0,138	371819,829
883,499	46950,84	6603,626	10,575	910,893	3867,364	2506,178	0,021	0,228	236613,385
901,560	47423,95	62074,883	121,948	96502,611	7692,308	14474,694	0,202	0,117	979500,674
923,540	47993,36	86773,828	172,613	121773,067	6872,009	24079,556	0,282	0,134	2682914,050
929,594	48149,01	21136,716	40,178	51778,685	7356,517	5162,490	0,069	0,126	132911,609
937,556	48352,93	4421,375	6,315	708,187	6449,857	974,379	0,014	0,145	90274,943
939,521	48403,10	11228,725	20,128	6332,712	6320,777	3140,857	0,037	0,149	187419,811
942,586	48481,30	8576,365	14,763	2633,430	6123,108	2370,592	0,028	0,154	142621,483
951,572	48709,78	30872,644	60,464	35194,799	6328,871	9603,550	0,100	0,150	922838,157
963,675	49015,83	4502,466	6,568	393,065	6647,504	1007,342	0,015	0,145	181505,412
964,573	49038,45	11687,350	21,342	9257,630	5605,584	3926,816	0,038	0,172	170787,406
967,553	49113,48	5054,360	7,707	888,849	5513,453	1431,236	0,016	0,175	121472,768
1023,647	50504,60	5855,907	9,975	1589,935	5720,599	1816,556	0,019	0,179	100825,127
1029,715	50652,76	8564,112	15,920	9627,184	7904,820	2062,686	0,028	0,130	37698,549
1051,696	51185,85	45085,447	97,200	97199,585	7347,142	14172,840	0,147	0,143	952479,642

1067,674	51569,87	8901,344	17,201	5450,478	6842,704	2698,024	0,029	0,156	85161,412
1079,729	51857,68	5328,793	9,363	1633,557	6697,789	1540,628	0,017	0,161	77983,348
1105,763	52473,78	4790,758	8,146	833,517	4682,300	1928,089	0,016	0,236	132301,510
1125,783	52942,64	13117,420	27,623	26514,163	8663,606	3516,861	0,043	0,130	68819,562
1147,765	53452,65	20630,235	45,879	42274,832	7616,449	6958,406	0,067	0,151	299670,224
1175,799	54096,02	3908,867	6,222	668,373	4559,868	1527,752	0,013	0,258	93479,605
1319,926	57288,45	4471,025	8,918	2089,588	8386,560	1259,386	0,015	0,157	42788,515
1341,904	57759,58	9398,951	22,836	15372,154	9272,044	3206,801	0,031	0,145	84334,092
1363,895	58227,14	12107,030	30,701	16620,856	7678,600	5321,607	0,039	0,178	193245,332
1379,869	58564,40	3858,690	7,533	1530,160	6568,645	1548,420	0,013	0,210	46853,523
1455,940	60144,28	15191,259	42,143	45440,745	9291,147	6386,628	0,049	0,157	188330,513
1483,970	60715,96	7448,470	19,208	6010,799	8289,182	3311,044	0,024	0,179	94600,015
1543,957	61921,45	39077,177	122,005	125771,419	9598,620	18619,239	0,127	0,161	998682,755
1557,973	62199,70	4312,767	9,735	1191,602	8126,883	1787,504	0,014	0,192	87460,505
1559,943	62238,71	4517,674	10,414	1210,695	7706,418	2003,049	0,015	0,202	99936,954
1571,990	62476,74	20004,463	61,288	32330,143	8812,405	10460,893	0,065	0,178	591319,658
1587,969	62791,03	3395,682	6,899	1333,139	7200,231	1443,800	0,011	0,221	40884,096
1600,038	63027,38	3676,955	7,923	791,313	5419,759	2169,606	0,012	0,295	124843,889
1631,977	63648,54	27308,229	89,310	124075,402	10028,319	13657,442	0,089	0,163	460932,544
1640,060	63804,77	3745,675	8,172	608,068	5132,954	2293,390	0,012	0,320	138872,955
1646,003	63919,41	3475,582	7,256	1180,051	6929,230	1613,764	0,011	0,238	52257,456
1647,967	63957,23	4053,797	9,275	1634,263	7767,205	1847,096	0,013	0,212	56249,249
1660,013	64188,79	17189,693	55,757	27763,222	9126,578	9493,643	0,056	0,182	478294,946
1688,047	64724,45	3781,097	8,563	1597,921	8169,875	1637,014	0,012	0,207	47621,204
1728,067	65481,46	4260,282	10,746	2027,682	5683,998	2985,295	0,014	0,304	76307,576
1756,096	66006,42	3401,929	7,697	3089,584	6355,449	1983,495	0,011	0,276	25533,316
1768,182	66231,50	5168,141	14,635	2301,366	8016,511	2960,535	0,017	0,221	94851,615
1856,196	67847,76	6605,751	21,950	9200,004	9701,929	3832,656	0,022	0,191	77067,653
1884,229	68354,43	3258,354	8,917	2855,826	8727,658	1719,222	0,011	0,216	26162,373
2080,375	71798,72	3290,910	12,782	3363,552	8556,659	2240,597	0,011	0,243	34194,721
2168,384	73291,05	4272,656	20,077	2947,220	9439,219	3050,270	0,014	0,230	77917,658
2196,429	73760,17	2477,129	10,099	1630,359	7567,274	1837,998	0,008	0,290	39476,627
2260,438	74819,79	2020,151	8,006	1130,380	7356,823	1459,265	0,007	0,307	33628,738
2348,457	76252,60	2180,340	10,373	2101,270	6595,380	1980,233	0,007	0,356	28731,379
2376,494	76703,31	1975,022	9,201	1692,652	6483,677	1738,356	0,006	0,367	26871,336
2436,466	77658,56	1513,468	6,522	2037,206	7476,048	1051,919	0,005	0,326	9654,383
2464,492	78100,90	1471,556	6,388	1688,785	7944,529	957,463	0,005	0,310	10069,335

List of MALDI-TOF MS mass peaks of culture medium

Table S7. List of MALDI-TOF mass peaks of culture medium used for *C. acnes* cultivation - thioglycollate-soy broth (TS).

m/z	time	Intens.	SN	Quality Fac.	Res.	Area	Rel. Intens.	FWHM	Chi^2
711,400	42173,21	5895,899	10,160	1434,793	7227,609	962,175	0,041	0,098	112881,501
733,382	42813,59	4979,054	8,424	1678,425	7305,451	822,477	0,035	0,100	68174,208
760,846	43600,28	3896,066	6,450	1875,371	7932,104	639,650	0,027	0,096	32079,231
838,424	45748,41	4025,972	6,357	2749,994	7376,511	781,373	0,028	0,114	25793,460
843,394	45882,57	6395,173	10,367	2330,654	6894,298	1408,550	0,045	0,122	103016,830
860,409	46338,84	19582,853	32,642	38133,217	7878,912	3986,069	0,137	0,109	102508,143
865,437	46472,80	20773,579	34,599	37916,610	7433,242	4438,285	0,145	0,116	140165,980
867,454	46526,42	7836,228	12,694	4110,087	8248,040	1509,728	0,055	0,105	70996,065
876,384	46763,12	5333,059	8,401	2103,617	7483,293	1154,055	0,037	0,117	67925,989
881,410	46895,79	6739,184	10,690	250,867	7737,191	1393,555	0,047	0,114	988672,195
882,399	46921,86	28104,473	46,622	19191,921	7367,165	6428,297	0,196	0,120	1063451,518
889,468	47107,72	9346,775	15,023	13868,059	7235,269	2105,318	0,065	0,123	33843,456
898,372	47340,79	12009,400	19,463	6153,021	7080,546	2961,417	0,084	0,127	126783,295
911,446	47680,92	143361,087	238,375	116364,179	7368,268	34782,882	1,000	0,124	9144060,241
927,423	48093,25	42507,686	70,005	112401,349	7845,870	9832,188	0,297	0,118	460837,320
933,433	48247,44	125315,449	207,560	107244,026	7109,962	32824,820	0,874	0,131	7721369,223
949,412	48654,96	60134,661	98,808	98453,419	7620,871	14888,343	0,419	0,125	1489249,330
965,385	49058,91	5999,444	9,079	1146,436	5395,933	2208,142	0,042	0,179	199706,557
982,508	49488,22	6658,393	10,181	2184,141	7706,603	1545,226	0,046	0,127	111347,186
987,538	49613,62	8452,453	13,159	5127,279	8091,468	1924,601	0,059	0,122	86013,391
1004,498	50034,10	4286,370	6,262	226,103	7084,415	1091,524	0,030	0,142	443770,060
1009,525	50158,06	5164,482	7,713	157,170	7210,130	1337,923	0,036	0,140	971181,683
1024,531	50526,22	6851,250	10,413	958,024	7298,323	1807,610	0,048	0,140	292329,245
1027,510	50598,98	4570,006	6,661	263,869	7318,294	1143,925	0,032	0,140	457579,989
1033,525	50745,58	6607,376	9,955	490,186	7433,880	1715,407	0,046	0,139	555562,942
1046,523	51060,90	7275,432	11,017	2459,246	7628,100	1900,856	0,051	0,137	127684,854
1062,498	51445,78	4299,074	6,153	575,830	6658,374	1376,499	0,030	0,160	185744,704
1073,516	51709,54	11502,873	17,975	6049,356	7804,268	3224,811	0,080	0,138	192785,040
1089,520	52090,26	5578,500	8,272	493,451	7009,129	1669,145	0,039	0,155	402358,192
1095,514	52232,12	7093,830	10,743	1672,970	7094,595	2224,536	0,049	0,154	195841,767
1105,539	52468,53	12913,466	20,269	4063,476	7969,398	3709,495	0,090	0,139	455491,843
1111,519	52609,03	4304,999	6,180	649,063	5789,987	1636,823	0,030	0,192	187413,407
1122,560	52867,45	32694,837	52,913	63025,491	8000,120	9843,736	0,228	0,140	418706,880
1127,531	52983,38	8986,971	13,909	2745,777	7735,504	2715,076	0,063	0,146	200682,076
1138,545	53239,34	9600,982	14,928	2686,591	7706,539	2968,660	0,067	0,148	215032,429
1144,550	53378,37	58270,714	95,364	23535,541	7316,625	20487,220	0,406	0,156	2320535,087
1160,532	53746,62	23116,078	37,478	6689,882	7301,416	8258,094	0,161	0,159	590820,495
1257,647	55931,58	5617,918	8,886	1067,214	8097,053	1868,204	0,039	0,155	209444,147
1284,639	56523,71	4365,436	6,829	871,436	8233,252	1432,698	0,030	0,156	147159,067
1306,630	57001,51	5001,255	8,128	1409,227	7234,138	2079,242	0,035	0,181	135041,712

1385,711	58687,26	6508,172	11,598	4993,162	8538,049	2485,164	0,045	0,162	65612,358
1401,698	59022,13	3654,622	6,254	852,856	8149,401	1470,502	0,025	0,172	121474,677
1986,028	70163,34	1917,093	8,807	3607,096	9104,308	1215,252	0,013	0,218	13012,040

List of MALDI-TOF MS mass peaks of norharmane matrix

Table S8. List of MALDI-TOF mass peaks of norharmane matrix.

m/z	time	Intens.	SN	Quality Fac.	Res.	Area	Rel. Intens.	FWHM	Chi^2
704,323	41964,96	3091,291	10,287	442,354	3245,918	1247,570	0,099	0,217	152038,434
707,394	42055,46	2229,978	7,425	353,553	1727,743	1722,007	0,072	0,409	174145,978
712,164	42195,65	3248,755	10,841	669,599	3404,233	1311,067	0,105	0,209	119394,238
715,055	42280,38	1939,669	6,480	504,103	2808,828	985,905	0,062	0,255	70748,476
737,267	42925,76	6234,696	20,972	2895,733	4372,003	2040,564	0,201	0,169	83924,308
779,228	44118,88	3239,626	11,096	1174,869	3431,275	1485,503	0,104	0,227	74549,634
802,347	44762,45	2618,820	9,072	609,207	5990,646	694,667	0,084	0,134	66178,799
803,355	44790,29	11642,515	40,322	30613,840	5581,332	3223,162	0,375	0,144	63947,377
810,220	44979,48	2376,488	8,257	725,711	5165,179	739,564	0,076	0,157	50046,214
823,235	45335,97	3108,973	10,869	106,245	2822,835	1895,019	0,100	0,292	1264209,697
825,330	45393,07	31081,363	108,681	110142,313	5400,757	9483,886	1,000	0,153	337147,920

MALDI-TOF MS lipid profiles of culture medium and matrix

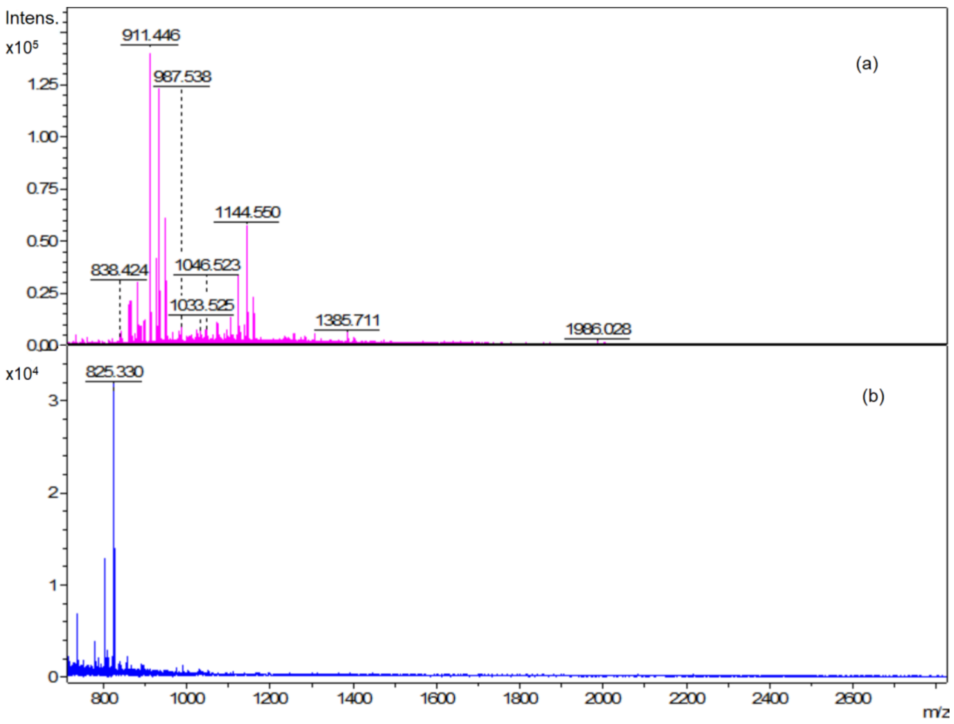

Figure S3. Positive ion MALDI-TOF MS spectra of lipid extract of culture medium used for *C. acnes* cultivation - thioglycollate-soy broth (a), and norharmane matrix (b).
